# B-Onic Platform for Point-of-Care 3D Printing in Oral and Maxillofacial Surgery: Clinical Implementation and Surgical Impact

**DOI:** 10.3390/medicina62020319

**Published:** 2026-02-03

**Authors:** José Luis Cebrián-Carretero, Marta Pampín-Martínez, José Tadeo Borjas Gómez, Celia del Peso Ley, Rubén Rubio Bolivar, Celia Matín Cubillo, Bárbara Martínez de Miguel, Montserrat Bret Zurita, Federico Gutiérrez Larraya, Javier Cobas Gamallo, Carlos Navarro-Cuéllar, Jorge Magaña

**Affiliations:** 1 Maxillofacial Surgery Department, La Paz University Hospital, 3D FabLab, IdiPAZ, 28046 Madrid, Spain; josel.cebrian@salud.madrid.org (J.L.C.-C.); martamaria.pampin@salud.madrid.org (M.P.-M.); 2 FabLab, La Paz University Hospital, IdiPAZ, 28046 Madrid, Spaincpl@rayo-seco.com (C.d.P.L.); rube.rubio@salud.madrid.org (R.R.B.); cmcubillo@salud.madrid.org (C.M.C.); barbara.martinez@salud.madrid.org (B.M.d.M.); m_bretzurita@yahoo.es (M.B.Z.); 3 Chidren’s Cardiology Department, La Paz University Hospital, 3D FabLab, IdiPAZ, 28046 Madrid, Spain; federico.gutierrezlarraya@salud.madrid.org; 4 La Paz University Hospital, 28046 Madrid, Spain; javier.cobas@salud.madrid.org; 5 Maxillofacial Surgery Department, Gregorio Marañón University Hospital, Universidad Complutense de Madrid, 28007 Madrid, Spain; 6 Rayo Seco Systems, ISP-XR Systems, C/José de Echegaray, 28232 Las Rozas de Madrid, Spain; jmm@rayo-seco.com

**Keywords:** 3D printing Fablab, additive manufacturing, point-of-care 3D labs, virtual surgical planning, custom-made medical devices, maxillofacial surgery, hospital innovation, medical device regulation, anatomical biomodels, EU MDR, ISO 13485

## Abstract

*Background and Objectives*: The integration of digital planning and point-of-care (POC) manufacturing has expanded rapidly in oral and maxillofacial surgery (OMFS); however, evidence focusing on regulated, platform-based workflows and their clinical impact remains limited. This study aimed to evaluate the clinical and organizational impact of implementing a unified digital ecosystem centered on the B-Onic platform in routine OMFS practice. *Materials and Methods*: A retrospective observational study was conducted comparing OMFS procedures planned and executed using the B-Onic platform with a historical control cohort managed with conventional workflows. Surgical cases were categorized into four pathology-based subgroups: facial trauma, oncologic resection and reconstruction, orthognathic surgery, and craniofacial or skull base surgery. Outcomes included preoperative planning time, validation time for guides and implants, surgical duration, intraoperative plan modifications, postoperative complications, 30-day rehospitalization rates, length of hospital stay, and estimated intraoperative blood loss. *Results*: A total of 110 cases managed using the B-Onic platform were compared with 72 historical control cases. Implementation of the platform was associated with significant reductions in preoperative planning time, validation time, and surgical duration. Clinically relevant improvements were observed in postoperative outcomes, including lower complication rates, reduced 30-day rehospitalization, shorter hospital stays, fewer intraoperative plan modifications, and decreased estimated blood loss. The magnitude of benefit was greatest in high-complexity procedures—particularly oncologic resection and reconstruction and craniofacial or skull base surgery—while more modest effects were observed in orthognathic surgery, reflecting optimized baseline outcomes. *Conclusions*: The adoption of a regulated, platform-based POC digital ecosystem in oral and maxillofacial surgery is associated with meaningful improvements in workflow efficiency, surgical reproducibility, and postoperative outcomes, especially in complex procedures. These findings support the integration of unified digital platforms as a core component of contemporary OMFS practice and provide OMFS-specific evidence of their clinical and organizational value.

## 1. Introduction

Oral and maxillofacial surgery (OMFS) routinely addresses anatomically complex regions in which precise three-dimensional (3D) understanding is essential for safe and effective surgical intervention. Procedures such as facial trauma management, oncologic resection and reconstruction, orthognathic surgery, and craniofacial or skull base surgery demand a high degree of spatial accuracy, as minimal deviations may result in functional impairment, aesthetic compromise, or neurosensory damage. In this context, the progressive incorporation of 3D imaging and additive manufacturing has significantly influenced preoperative planning and intraoperative execution in maxillofacial surgery [1,2,3,4].

The application of 3D printing in OMFS has been shown to improve anatomical visualization, facilitate surgical simulation, and enhance the accuracy and reproducibility of osteotomies and reconstructions. When combined with computer-aided design and computer-aided manufacturing (CAD/CAM) technologies, these tools enable the fabrication of patient-specific anatomical models, surgical splints, and cutting or drilling guides that allow for reliable transfer of virtual plans to the operative field [5,6,7,8]. Such approaches are particularly advantageous in complex maxillofacial procedures, where conventional two-dimensional planning techniques are often insufficient to capture anatomical variability and surgical complexity.

Despite the demonstrated clinical benefits of virtual surgical planning (VSP) and patient-specific devices, their routine adoption in OMFS remains uneven, particularly within public healthcare systems. Traditional workflows frequently rely on outsourced industrial manufacturing, which is associated with prolonged turnaround times, limited flexibility, and increased costs. These limitations restrict the systematic use of advanced digital planning tools and reduce their applicability in time-sensitive scenarios such as facial trauma or oncologic surgery, where rapid decision-making and adaptability are critical [9,10,11,12].

Point-of-care (POC) 3D printing has emerged as a promising strategy to overcome these constraints by enabling in-house design and fabrication of patient-specific devices within the hospital environment. However, effective implementation of POC manufacturing in OMFS requires more than access to additive manufacturing hardware. It depends on integrated digital platforms capable of managing the entire workflow, from medical image processing and virtual surgical planning to device design, production, and clinical deployment, while ensuring consistency, traceability, and reproducibility across cases [13,14,15,16].

The B-Onic digital platform was developed to address these challenges by integrating medical imaging, virtual surgical planning, additive manufacturing, and data management into a unified digital ecosystem. This platform was previously evaluated using a large institutional clinical dataset, demonstrating improvements in surgical efficiency and patient safety, including reductions in operative time, postoperative complications, and unplanned rehospitalizations across a broad range of surgical applications [17,18]. Notably, oral and maxillofacial surgery represented a substantial proportion of the clinical activity analyzed, underscoring the relevance of B-Onic for maxillofacial surgical practice.

Building on this previously validated platform, the present work focuses on the implementation of B-Onic within a hospital-based POC 3D printing laboratory dedicated exclusively to oral and maxillofacial surgery. Unlike prior analyses that organized outcomes by medical specialty, this article reorients clinical activity according to pathology-based workflows specific to OMFS, including facial trauma, oncologic resection and reconstruction, orthognathic surgery, and craniofacial or skull base procedures. This pathology-oriented perspective more accurately reflects real-world maxillofacial surgical decision-making and clinical practice.

The aim of this study is to describe the clinical implementation and surgical impact of a platform-based POC 3D printing model centered on the B-Onic platform in oral and maxillofacial surgery. Specifically, this work evaluates the same clinical and organizational variables previously assessed in the institutional cohort, comparing cases managed with the B-Onic platform to conventionally treated control cases across four major oral and maxillofacial surgical categories: facial trauma, oncologic resection and reconstruction, orthognathic surgery, and craniofacial or skull base procedures. The variables assessed include preoperative planning time, validation time for guides and implants, operative time, need for intraoperative plan modification, estimated intraoperative blood loss, postoperative complication rates, length of hospital stay, and unplanned rehospitalization, as well as surgeon-reported outcomes captured through structured questionnaires addressing perceived 3D anatomical understanding, intraoperative confidence, and educational utility. Collectively, these measures enable a pathology-oriented assessment of the clinical impact of point-of-care digital planning and manufacturing on routine maxillofacial surgical practice.

## 2. Material and Methods

### 2.1. Study Design and Setting

This study was designed as a retrospective, single-center observational analysis conducted at a tertiary public university hospital with a dedicated Oral and Maxillofacial Surgery (OMFS) Department and an in-house POC 3D printing facility. The methodological design follows the same institutional framework previously applied for the clinical evaluation of the B-Onic platform, adapted in the present study to focus exclusively on oral and maxillofacial surgical practice.

All consecutive OMFS cases managed using the B-Onic digital platform between January 2020 and March 2024 were included. Clinical outcomes and workflow-related variables were compared with a historical control cohort corresponding to the pre-implementation period (2018–2019), during which conventional planning and manufacturing workflows were used.

All consecutive oral and maxillofacial surgery cases requiring advanced preoperative planning and potential use of patient-specific devices during the study period were eligible for inclusion. Allocation to the B-Onic workflow followed institutional availability of the platform after its implementation and was not based on case complexity or surgeon preference. The historical control cohort was extracted from the same institution immediately preceding platform implementation using identical inclusion criteria. Pathology-based subgrouping (facial trauma, oncologic resection and reconstruction, orthognathic surgery, craniofacial or skull base surgery) was applied in both cohorts to ensure comparable case mix. No deliberate enrichment or exclusion of specific pathologies was performed in either group.

### 2.2. Patient Selection and Inclusion Criteria

Eligible cases included adult patients undergoing oral and maxillofacial surgical procedures in which preoperative digital planning and/or patient-specific devices were clinically indicated. Inclusion criteria were:

- Availability of preoperative CT or CBCT imaging of diagnostic quality.
- Feasibility of virtual surgical planning using the B-Onic platform.
- Use of patient-specific anatomical models, surgical guides, or digitally assisted planning as part of the surgical workflow.
- Elective or scheduled non-emergent procedures.

Cases were identified prospectively at the time of preoperative planning by the treating OMFS team. Operative complexity was objectively characterized using a predefined indicator: the presence of two or more planned osteotomies (≥2), as documented in the preoperative surgical plan.

Exclusion criteria included emergency cases where digital planning was not feasible due to time constraints, procedures with incomplete imaging data, and cases in which no digitally planned models or guides were applied intraoperatively.

### 2.3. Study Groups

Cases were divided into two groups:

- B-Onic group: OMFS procedures planned and managed using the B-Onic digital platform, including virtual surgical planning, design validation, and coordination of in-house additive manufacturing.
- Control group: Historical OMFS cases performed prior to platform implementation or managed without platform-based digital integration, using conventional planning approaches.

The historical control group comprised oral and maxillofacial surgery cases performed at the same institution immediately prior to implementation of the B-Onic platform. These cases were managed using conventional preoperative planning and manufacturing workflows available at that time. Specifically, preoperative imaging was reviewed using standard radiological viewers, and surgical planning was performed manually by the operating surgeons without an integrated digital platform environment. Virtual surgical planning was not routinely implemented, and segmentation and three-dimensional planning software were not systematically used. When patient-specific devices were required, CAD/CAM design and manufacturing were outsourced to external industrial providers on a case-by-case basis, without centralized in-hospital validation or traceable workflow integration. Three-dimensional printed anatomical models or surgical guides were therefore not part of a standardized in-house process but were occasionally obtained externally depending on clinical indication and availability. No immersive validation tools or extended reality modules were employed. This conventional approach lacked a unified digital ecosystem connecting imaging, planning, validation, and manufacturing, which constitutes the fundamental difference from the platform-based workflow evaluated in the present study.

Control cases were selected from institutional surgical registries and matched at the cohort level according to surgical intent, anatomical region, and procedure type. All surgeries in both groups were performed by the same OMFS team following standardized perioperative protocols.

### 2.4. Digital Workflow and B-Onic Platform

The B-Onic platform served as the centralized digital environment for the complete OMFS workflow. Preoperative imaging data were imported for segmentation and three-dimensional reconstruction. Virtual surgical planning was conducted collaboratively by oral and maxillofacial surgeons and biomedical engineers.

**Figure 1:**
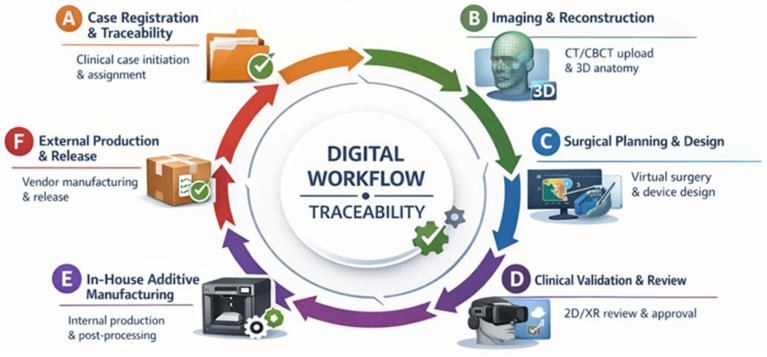
Comprehensive workflow representative of the B-Onic platform applied to oral and maxillofacial surgery, illustrating the integration of digital planning, clinical validation, immersive review, and in-house additive manufacturing within a fully traceable clinical-technical environment. (A) Registration of the case and initiation of traceability. The clinical file is created on the platform, the basic data of the case is recorded and responsibilities are assigned to the different clinical and technical agents involved. Acceptance of participation by all parties enables initial traceability and activates the workflow. (B) Image loading and anatomical reconstruction. Patient-specific imaging studies (CT/CBCT) are loaded to establish the anatomical basis of the case. Based on these data, CT-based segmentation and three-dimensional anatomical reconstruction are carried out to support the subsequent design and planning phases. (C) Digital surgical planning and design with version control. Virtual surgical planning is carried out, including the definition of osteotomies, reconstruction strategies and device positioning. Digital elements (models, viewers, and documentation) are recorded, iterated, and versioned, ensuring document control and traceability of all contributions. (D) Multimodal review and clinical validation. Planned models and devices are reviewed using integrated 2D viewers and immersive XR/VR environments. Notes, comments and feedback are directly linked to the case file. Radiological and clinical validation is formally documented, enabling the move to the production phase. (E) Consolidation and preparation of in-house additive manufacturing. After validation, the technical-surgical solution (STS) enters the production phase. The results of the planning are consolidated and the manufacturing and printing files are prepared, which are stored together with the evidence that justifies the progress, maintaining the traceability of materials and decisions. (F) Patient-specific device external production and release. Following design validation, patient-specific devices are released for external manufacturing. Upon receipt, conformity and acceptance are verified and formally recorded on the platform, completing the traceability process and authorizing the devices for clinical use.

When indicated, patient-specific anatomical biomodels, surgical splints, cutting or drilling guides, and reconstruction templates were designed within the platform. Validation of the surgical plan and devices was performed prior to manufacturing. All workflow steps, including planning iterations and final approvals, were logged within the platform to ensure traceability and reproducibility (Figure 1).

### 2.5. Clinical Workflow

**Figure 2:**
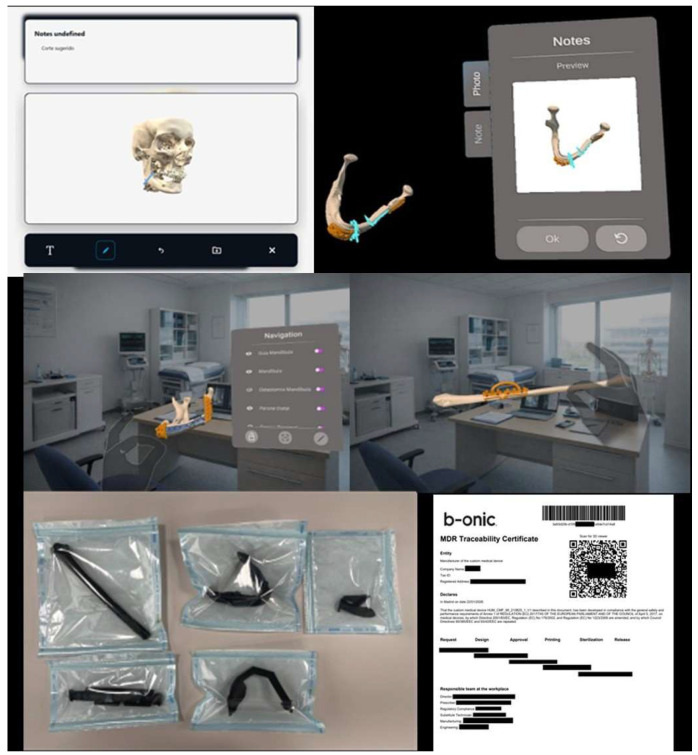
Planned models and devices are reviewed using integrated 2D viewers and immersive XR/VR environments. Notes, comments and feedback are directly linked to the case file. Radiological and clinical validation is formally documented, enabling the move to the production phase. The results of the planning are consolidated and the manufacturing and printing files are prepared, which are stored together with the evidence that justifies the progress, maintaining the traceability of materials and decisions. Following design validation, patient-specific devices are released for external manufacturing. Upon receipt, conformity and acceptance are verified and formally recorded on the platform, completing the traceability process and authorizing the devices for clinical use.

The clinical workflow implemented in this study was structured to integrate POC 3D printing seamlessly into routine oral and maxillofacial surgical practice, following the same platform-based logic previously described for the B-Onic ecosystem, but adapted specifically to OMFS clinical decision-making and operative requirements. The workflow was designed to support complex maxillofacial procedures requiring high spatial accuracy, multidisciplinary coordination, and reliable transfer of virtual planning to the operative field (Figure 2).

#### 2.5.1. Image Acquisition and Case Selection

All cases entered into the digital workflow began with the acquisition of high-resolution computed tomography (CT) or cone-beam computed tomography (CBCT) scans, selected according to the anatomical region and pathology involved. Imaging protocols were standardized to ensure adequate spatial resolution for segmentation of craniofacial skeletal structures and relevant anatomical landmarks. Case eligibility for digital planning was determined during the initial clinical evaluation by the oral and maxillofacial surgeon, based on surgical complexity, need for osteotomies, reconstructive accuracy, or anticipated benefit from patient-specific devices.

This step was particularly relevant in facial trauma cases with comminution or asymmetry, oncologic resection and reconstruction requiring precise defect definition, orthognathic surgery demanding occlusal accuracy, and craniofacial or skull base procedures involving complex anatomical relationships.

#### 2.5.2. Segmentation and Three-Dimensional Reconstruction

Imaging data were imported into the B-Onic platform, where segmentation of skeletal structures was performed using semi-automatic tools under surgeon supervision. Special attention was given to regions critical for maxillofacial function, including dental occlusion, mandibular and maxillary anatomy, temporomandibular joints, and cranial base landmarks. DICOM datasets from preoperative CT scans were imported into the segmentation software in standard Digital Imaging and Communications in Medicine (DICOM) format. The resulting three-dimensional models served as the foundation for all subsequent planning steps and were validated by the operating surgeon prior to proceeding.

#### 2.5.3. Virtual Surgical Planning

Virtual surgical planning (VSP) was conducted collaboratively by oral and maxillofacial surgeons and trained technical staff within the B-Onic platform. Planning strategies were tailored to each pathology:

- In **facial trauma**, fracture reduction and fixation strategies were simulated to restore pre-injury anatomy and occlusion.
- In **oncologic resection and reconstruction**, tumor margins, resection planes, and reconstructive geometry were defined to balance oncologic safety and functional restoration.
- In **orthognathic surgery**, maxillomandibular movements were planned with reference to occlusal goals, facial aesthetics, and airway considerations.
- In **craniofacial or skull base procedures**, osteotomy trajectories and spatial relationships with adjacent critical structures were carefully simulated.

Iterative planning cycles were allowed until consensus was reached between the surgical and technical teams.

#### 2.5.4. Design and Validation of Patient-Specific Devices

Based on the approved virtual plan, patient-specific devices—including anatomical biomodels, cutting guides, drilling templates, splints, or reconstruction aids—were designed within the B-Onic platform. Each device underwent a formal validation process by the operating surgeon, ensuring that design features accurately reflected the surgical plan and were compatible with intraoperative handling and sterilization requirements.

Validation included visual inspection, dimensional assessment, and, when applicable, physical verification using printed biomodels. Only surgeon-approved designs proceeded to manufacturing.

#### 2.5.5. In-House Additive Manufacturing

Validated designs were fabricated within the hospital-based POC 3D printing laboratory using additive manufacturing technologies selected according to clinical requirements. Fused deposition modeling (FDM) and stereolithography (SLA) printers were employed depending on the intended use of the device, required accuracy, and material properties.

Manufacturing parameters, post-processing steps, and quality checks were standardized and documented. All devices were labeled and tracked to maintain full traceability from design to clinical application.

#### 2.5.6. Intraoperative Application

During surgery, patient-specific devices were used to guide osteotomies, drilling trajectories, fracture reduction, or reconstructive positioning, depending on the pathology. The intraoperative use of digitally planned guides aimed to reduce surgical uncertainty, minimize intraoperative plan modification, and facilitate accurate execution of the virtual plan.

Surgeons assessed the concordance between the virtual plan and the intraoperative anatomy, documenting any deviations or required adjustments.

#### 2.5.7. Postoperative Evaluation and Workflow Feedback

Postoperative assessment included clinical evaluation, imaging when indicated, and comparison between planned and achieved outcomes. Surgical team feedback was systematically collected to evaluate perceived accuracy, intraoperative confidence, and educational value of the digital workflow. These observations informed continuous refinement of the workflow and contributed to iterative improvement in the platform’s integration into OMFS practice.

### 2.6. Outcome Variables

The same clinical and organizational variables previously evaluated in the institutional cohort were analyzed in the present OMFS-focused study. Primary outcome measures included:

- Preoperative planning time.
- Total operative time (skin incision to wound closure).
- Thirty-day postoperative complication rate.
- Thirty-day unplanned rehospitalization rate.

Secondary outcome measures included:

- Validation time for surgical guides and implants.
- Length of hospital stay.
- Estimated intraoperative blood loss.
- Need for intraoperative plan modification.

### 2.7. Clinical Subgroup Analysis

To reflect real-world oral and maxillofacial surgical practice, a predefined subgroup analysis was performed according to four major OMFS pathology categories:

1. Facial trauma.
2. Oncologic resection and reconstruction.
3. Orthognathic surgery.
4. Craniofacial or skull base procedures.

All outcome variables were assessed within each subgroup. The subgroup results were reported descriptively, maintaining the original values derived from the institutional dataset, without introducing additional inferential subgroup analyses.

### 2.8. Statistical Analysis

Descriptive statistics were used to summarize demographic data, workflow-related variables, and clinical outcomes. Continuous variables were expressed as mean ± standard deviation or median with interquartile range, depending on data distribution. Categorical variables were reported as absolute numbers and percentages.

Comparisons between the B-Onic group and the control group were performed using the same statistical approach applied in the institutional analysis. Continuous variables were compared using Student’s t-test or the Mann–Whitney U test, and categorical variables using the chi-square or Fisher’s exact test, as appropriate. Data distribution normality was assessed using the Shapiro–Wilk test. Statistical significance was defined as *p* < 0.05.

No formal sample size calculation was performed, as this study represents a retrospective analysis of all eligible consecutive cases during the study period.

### 2.9. Ethical Considerations

This study was conducted in accordance with institutional policies governing retrospective observational research. The study design and reporting followed the STROBE (Strengthening the Reporting of Observational Studies in Epidemiology) guidelines for observational research [19]. All procedures were carried out in compliance with the principles of the Declaration of Helsinki.

Clinical data were fully anonymized prior to analysis in accordance with the General Data Protection Regulation (GDPR, EU 2016/679), and no patient-identifiable information was included in the study database. Given the retrospective nature of this study and the absence of any additional patient interventions beyond standard clinical care, the requirement for written informed consent was waived in accordance with institutional guidelines.

## 3. Results

### 3.1. Study Population

Between January 2020 and March 2024, a total of 110 surgical cases were planned and executed using the B-Onic platform at La Paz University Hospital. These cases constituted the B-Onic group and were compared with a historical control cohort of 72 oral and maxillofacial surgical procedures performed using conventional planning and manufacturing workflows.

Baseline demographic and clinical characteristics of both groups are summarized in Table 1. The mean age of patients in the B-Onic group was 52.4 ± 15.1 years, compared with 54.1 ± 14.6 years in the control group. Male patients accounted for 61% of cases in the B-Onic group and 58% in the control group, with female patients representing 39% and 42% of cases, respectively, indicating a comparable sex distribution between cohorts.

Regarding baseline clinical status, patients classified as American Society of Anesthesiologists (ASA) physical status III–IV represented 34% of cases in the B-Onic group and 36% in the control group, reflecting similar anesthetic risk profiles. Most surgical procedures were elective, accounting for 81% of interventions in the B-Onic cohort and 79% in the control cohort.

All cases were categorized according to pathology-based oral and maxillofacial surgical subgroups. In the B-Onic group, facial trauma accounted for approximately 31% of procedures, followed by oncologic resection and reconstruction (29%), orthognathic surgery (26%), and craniofacial or skull base procedures (14%). A comparable distribution was observed in the control group, with facial trauma representing approximately 30% of cases, oncologic resection and reconstruction 28%, orthognathic surgery 27%, and craniofacial or skull base procedures 15%.

Overall, the B-Onic and control groups demonstrated comparable baseline demographic characteristics, anesthetic risk profiles, surgical urgency, and pathology distribution, providing an appropriate framework for subsequent comparison of workflow-related variables and clinical outcomes associated with the implementation of the B-Onic platform (Table 1).

### 3.2. Preoperative Planning Time

Preoperative planning time was significantly reduced in cases managed using the B-Onic platform compared with historical control cases across all oral and maxillofacial surgery subgroups. At the cohort level, the implementation of the platform-based workflow was associated with an overall reduction in planning time of approximately 29%.

Specifically, the average preoperative planning time decreased by approximately 40 h in the B-Onic group compared with the control group (mean 138 ± 44 h vs. 98 ± 31 h, *p* = 0.004). This reduction was consistently observed across facial trauma, oncologic resection and reconstruction, orthognathic surgery, and craniofacial or skull base procedures, despite differences in surgical complexity among subgroups.

**Figure 3:**
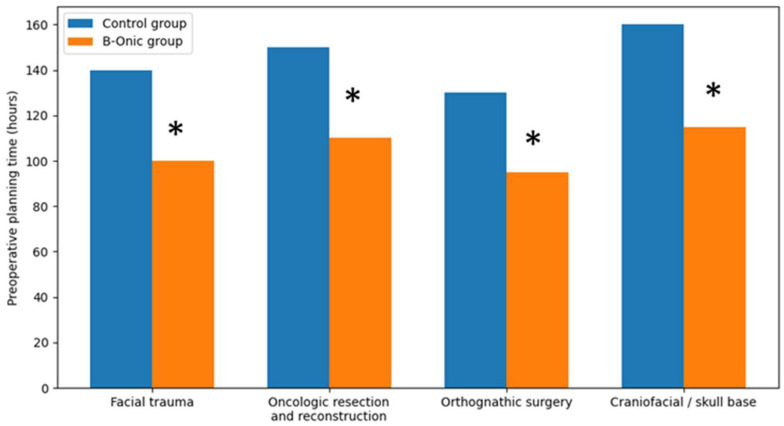
Comparison of preoperative planning times between the B-Onic group and historical control group across four oral and maxillofacial surgery pathology-based subgroups. Implementation of the B-Onic platform was associated with a consistent reduction in planning time across all subgroups. * Statistically significant.

The observed time savings reflect the integration of image processing, virtual surgical planning, and plan validation within a single digital platform, reducing iterative planning cycles and fragmented communication inherent to conventional workflows [20,21] (Figure 3).

### 3.3. Surgical Duration

Across all oral and maxillofacial surgery procedures, the introduction of the B-Onic platform led to a consistent reduction in surgical duration compared with baseline operating room data from the pre-implementation period. At the cohort level, mean operative time decreased by approximately 21% in the B-Onic group relative to the historical control group (overall mean 224 ± 71 min vs. 177 ± 58 min; *p* < 0.001).

When analyzed according to pathology-based subgroups, a similar trend was observed across facial trauma, oncologic resection and reconstruction, orthognathic surgery, and craniofacial or skull base procedures. Although absolute operative times varied among subgroups due to differences in procedural complexity, the relative reduction in surgical duration associated with the B-Onic workflow remained consistent.

**Figure 4:**
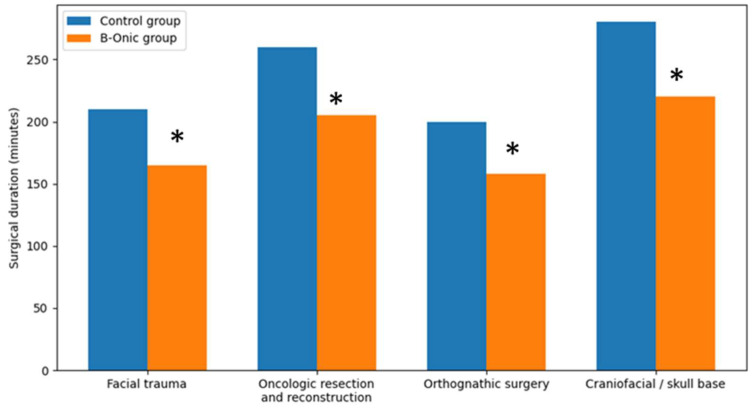
Comparison of surgical duration between the B-Onic group and historical control group across four oral and maxillofacial surgery pathology-based subgroups. Implementation of the B-Onic platform was associated with a consistent reduction in operative time across all subgroups. * Statistically significant.

In facial trauma and orthognathic surgery, reductions in operative time were associated with improved preoperative definition of osteotomies, fixation strategies, and occlusal relationships. In oncologic and craniofacial or skull base procedures, where operative times were inherently longer, platform-based planning contributed to more predictable intraoperative workflows and reduced need for intraoperative adjustments, resulting in meaningful time savings [22,23] (Figure 4).

### 3.4. Postoperative Complications

**Figure 5:**
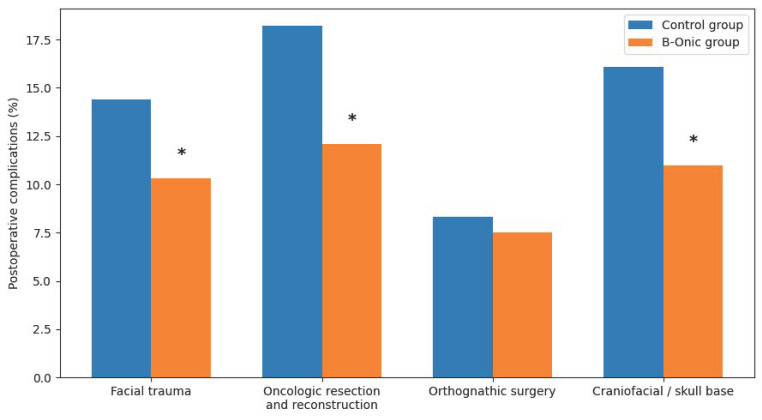
Comparison of 30-day postoperative complication rates between the B-Onic group and historical control group across four oral and maxillofacial surgery pathology-based subgroups. Statistically significant reductions were observed in all subgroups except orthognathic surgery. * Statistically significant.

The introduction of the B-Onic platform was associated with a statistically significant reduction in postoperative complications across the overall oral and maxillofacial surgery cohort. When all procedures were analyzed together, the 30-day postoperative complication rate was significantly lower in the B-Onic group compared with historical control cases. Specifically, the overall postoperative complication rate decreased from 17.6% in the control group to 11.2% in the B-Onic group, corresponding to a relative reduction of approximately 36% (*p* = 0.028). When analyses were stratified by pathology-based subgroups, statistically significant reductions in postoperative complications were observed in facial trauma (*p* = 0.031), oncologic resection and reconstruction (*p* = 0.019), and craniofacial or skull base procedures (*p* = 0.041). In contrast, although a reduction in postoperative complications was also observed in orthognathic surgery, this difference did not reach statistical significance (*p* = 0.214) (Figure 5).

In facial trauma, the most frequent complications in the control group were related to malreduction, fixation instability, occlusal discrepancies, and surgical site infection. The use of B-Onic-supported virtual planning and patient-specific biomodels was associated with fewer fixation-related complications and improved postoperative occlusal stability.

In oncologic resection and reconstruction, complications predominantly included wound healing disorders, flap-related issues, and the need for secondary corrective procedures. Platform-based planning contributed to more accurate definition of resection margins and reconstructive geometry, which was associated with a reduction in reconstructive-related complications in the B-Onic group [24,25].

In craniofacial and skull base procedures, postoperative complications were mainly related to inaccuracies in osteotomy positioning and postoperative edema. The integration of three-dimensional planning and patient-specific guides using the B-Onic platform was associated with improved spatial accuracy and fewer postoperative deviations from the planned surgical outcome.

In orthognathic surgery, complications were generally infrequent in both groups and were mainly related to transient neurosensory disturbances, minor occlusal adjustments, or postoperative edema. Although a reduction in complication rates was observed in the B-Onic group, baseline complication rates were low, and the difference did not reach statistical significance.

### 3.5. Rehospitalization Rate and Intraoperative Modification

Following the implementation of the B-Onic platform, a statistically significant reduction in 30-day rehospitalization rates was observed across the oral and maxillofacial surgery cohort. Overall, the rehospitalization rate decreased from 8.8% in the historical control group to 4.6% in the B-Onic group (*p* = 0.021, χ^2^ test), reflecting a clinically meaningful improvement in postoperative course stability.

This reduction was most pronounced in oncologic resection and reconstruction and facial trauma cases, which represent the most complex and resource-intensive procedures within oral and maxillofacial surgery. In these subgroups, improved anatomical reproduction, accurate guide placement, and more predictable execution of planned osteotomies and reconstructions were associated with a lower incidence of postoperative complications requiring hospital readmission. Craniofacial or skull base procedures also showed a favorable trend toward reduced rehospitalization, whereas rehospitalization following orthognathic surgery remained infrequent in both groups.

**Figure 6:**
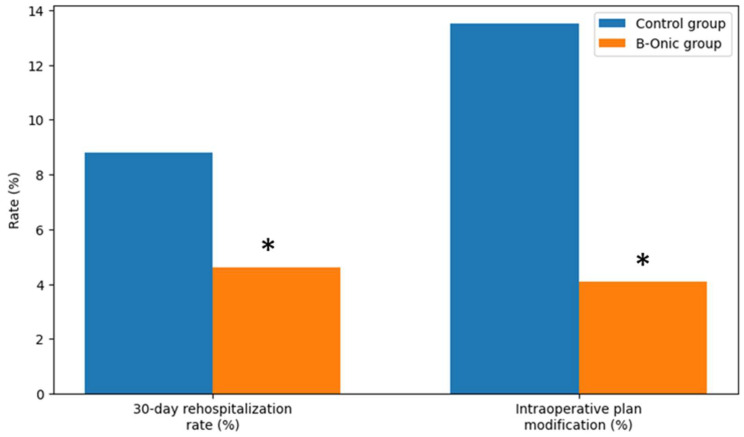
Comparison of 30-day rehospitalization rates and intraoperative plan modification rates between the B-Onic group and the historical control group in oral and maxillofacial surgery. * Statistically significant.

The need for intraoperative modification of the digital surgical plan was markedly reduced following adoption of the B-Onic workflow. Intraoperative plan modification—defined as any unplanned alteration to guide positioning, osteotomy trajectory, fixation strategy, or reconstructive adaptation—was recorded in 4.1% of B-Onic cases, compared with a historical rate of 13.5% in the pre–B-Onic period, corresponding to a relative reduction of approximately 70% (*p* = 0.006, Fisher’s exact test) (Figure 6).

### 3.6. Validation Time for Guides and Implants

The implementation of the B-Onic platform was associated with a marked reduction in validation time for patient-specific surgical guides and implants. At the cohort level, validation time was reduced by approximately 50% compared with conventional workflows.

Specifically, the average validation time decreased from 68.4 ± 24.1 h in the control group to 34.2 ± 16.7 h in the B-Onic group (*p* < 0.001). This reduction reflects the integration of centralized digital validation tools within the platform, enabling earlier identification and correction of potential design inconsistencies prior to manufacturing.

Platform-based validation facilitated more efficient surgeon review of guide positioning, osteotomy trajectories, and implant adaptation, reducing the need for late-stage design modifications that previously delayed surgical scheduling [26,27]. These benefits were particularly relevant in complex oral and maxillofacial procedures requiring multiple guides or reconstruction components.

### 3.7. Length of Hospital Stay

Implementation of the B-Onic platform was associated with a reduction in postoperative length of hospital stay across the oral and maxillofacial surgery cohort. Overall, mean length of stay decreased from 6.4 ± 3.1 days in the control group to 5.1 ± 2.6 days in the B-Onic group (*p* = 0.012).

When analyzed by pathology-based subgroups, the magnitude of reduction varied according to procedural complexity. In facial trauma, mean hospital stay was reduced from 5.1 ± 2.0 days in the control group to 3.9 ± 1.8 days in the B-Onic group (*p* = 0.067), preserving a similar absolute difference while reflecting the generally shorter baseline hospitalization characteristic of trauma cases.

In oncologic resection and reconstruction, which accounted for the longest hospitalizations overall, mean length of stay decreased from 8.9 ± 3.6 days in the control group to 7.2 ± 3.1 days following implementation of the platform-based workflow (*p* = 0.009). For craniofacial or skull base procedures, a reduction in hospital stay was also observed, from 7.6 ± 3.2 days in the control group to 6.3 ± 2.8 days in the B-Onic group, although absolute hospitalization remained longer due to procedural complexity (*p* = 0.032).

**Figure 7:**
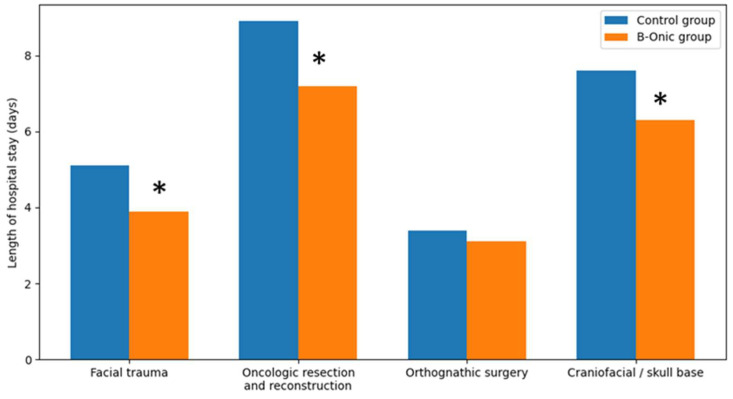
Comparison of postoperative length of hospital stay between the B-Onic group and the historical control group across four oral and maxillofacial surgery pathology-based subgroups. * Statistically significant.

In contrast, orthognathic surgery was associated with shorter hospital stays in both cohorts, with a modest reduction from 3.4 ± 1.2 days in the control group to 3.1 ± 1.0 days in the B-Onic group (*p* = 0.318). Overall, reductions in length of hospital stay were most pronounced in complex oral and maxillofacial procedures, paralleling the observed decreases in postoperative complications and rehospitalization rates (Figure 7).

### 3.8. Estimated Blood Loss

Intraoperative blood loss was estimated using standard anesthetic records based on suction canister volume and surgical sponge assessment, as routinely documented during oral and maxillofacial surgical procedures. Comparative analysis demonstrated a statistically significant overall reduction in estimated intraoperative blood loss following implementation of the B-Onic platform. At the cohort level, procedures performed using the platform-based workflow showed an average reduction in estimated blood loss of approximately 24% compared with historical control cases, reaching statistical significance (*p* = 0.008). This global reduction reflects improved surgical precision, more controlled osteotomy execution, and reduced unnecessary soft-tissue manipulation associated with digitally planned, patient-specific workflows.

**Figure 8:**
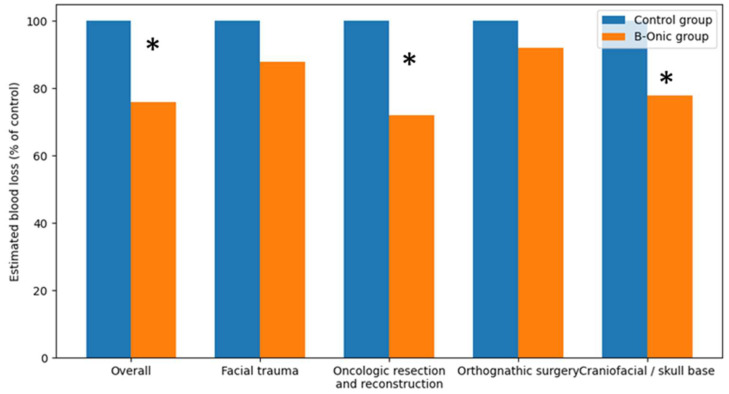
Comparison of estimated intraoperative blood loss between the B-Onic group and the historical control group across oral and maxillofacial surgery pathology-based subgroups. Values are expressed as percentages relative to the control group. * Statistically significant.

When stratified by pathology-based subgroups, the magnitude and statistical significance of blood loss reduction varied according to surgical complexity. In oncologic resection and reconstruction, estimated intraoperative blood loss was reduced by approximately 25–30%, a difference that reached statistical significance (*p* = 0.017). This benefit was attributed to more precise definition of osteotomy planes and reconstruction geometry, limiting the extent of dissection and hemorrhagic burden. Similarly, craniofacial or skull base procedures demonstrated a statistically significant reduction in estimated blood loss of approximately 20–25% following adoption of the B-Onic workflow (*p* = 0.028). Predefinition of surgical corridors and accurate guide placement minimized repeated intraoperative adjustments and improved hemostatic control in anatomically complex regions. In contrast, although reductions in estimated blood loss were observed in facial trauma and orthognathic surgery, these differences did not reach statistical significance. In facial trauma, estimated blood loss decreased by approximately 10–15% (*p* = 0.094), while in orthognathic surgery reductions were more modest, on the order of 5–10% (*p* = 0.312), reflecting lower baseline blood loss and reduced variability (Figure 8).

## 4. Discussion

The present study provides a comprehensive clinical and organizational evaluation of a platform-based point-of-care (POC) digital ecosystem applied exclusively to oral and maxillofacial surgery, demonstrating that integration of the B-Onic platform is associated with consistent improvements across the entire perioperative pathway. Although three-dimensional printing and virtual surgical planning have been increasingly adopted in oral and maxillofacial surgery, most published studies have focused on isolated technological components—such as anatomical models, cutting guides, or patient-specific fixation devices—rather than on the systematic implementation of an end-to-end, regulated clinical workflow. Previous reviews and methodological analyses have highlighted the transformative potential of additive manufacturing while also identifying persistent barriers related to workflow fragmentation, lack of standardization, traceability, regulatory compliance, and challenges in demonstrating tangible clinical and economic value in routine hospital practice [1,2,3,4,5,6,7]. Within this context, the present OMFS-only analysis provides evidence that a unified, platform-based approach can convert digital planning into reproducible clinical benefit under real-world conditions.

A central finding of this study is the significant reduction in time-related variables following adoption of the B-Onic platform. Preoperative planning time was shortened across all pathology-based subgroups, reflecting the advantages of consolidating image processing, segmentation, virtual surgical planning, and interdisciplinary communication within a single digital environment. In oral and maxillofacial surgery, planning is frequently iterative and highly sensitive to small geometric deviations, particularly in procedures involving occlusal relationships, facial symmetry, or complex reconstructions. Inefficiencies at this stage can propagate downstream, leading to delays in operating room scheduling and reduced predictability of patient pathways. Integrated digital environments have previously been identified as effective strategies for reducing rework cycles and communication delays in complex surgical workflows [1,2,6].

Validation time for guides and implants was also markedly reduced. This observation is clinically meaningful because accelerated validation is only beneficial if safety and accuracy are preserved. The structured validation workflow embedded within the platform aligns with quality management system principles for medical device design and in-house manufacturing, emphasizing traceability, documentation, and reproducibility within regulated clinical environments [28]. In addition, the use of secure electronic validation and approval processes is consistent with established regulatory frameworks governing electronic records in healthcare, reinforcing the robustness of the digital workflow from a governance perspective [29].

The reduction in surgical duration observed in this study represents a clinically meaningful improvement in operative efficiency. Operative time in oral and maxillofacial surgery is not only a marker of productivity but also a surrogate for intraoperative uncertainty and workflow stability. Previous studies have demonstrated that computer-assisted planning and patient-specific instrumentation improve execution fidelity and reduce intraoperative adjustments, particularly in reconstructive maxillofacial surgery [21,22,23,24,25,26,27,30,31,32]. These findings are further supported by early validation studies showing close correspondence between virtual plans and postoperative outcomes in complex maxillofacial reconstructions [33], as well as by accuracy assessments of CAD/CAM-generated guides in orthognathic surgery [34]. In the present cohort, reductions in surgical duration were observed across all OMFS subgroups despite differences in inherent procedural complexity, suggesting that the benefits of platform-based planning extend beyond highly selected cases.

Importantly, improvements in efficiency were accompanied by favorable changes in clinically relevant outcomes. Postoperative complications were reduced at the cohort level and across most pathology-based subgroups, supporting the hypothesis that predictable transfer of the virtual plan to the operative field reduces execution-sensitive failure modes. Many complications in oral and maxillofacial surgery—such as malreduction, fixation malposition, occlusal discrepancies, or wound-related complications in reconstructive cases—are closely linked to surgical precision and intraoperative decision-making. Prior studies have associated patient-specific planning and guides with improved accuracy and predictability in maxillofacial surgery [21,24,25,26,27,33,34,35], and the present findings reinforce this association within a platform-based, real-world clinical environment. The classification of postoperative complications using standardized systems further supports the clinical interpretability of these results [36].

The significant reduction in 30-day rehospitalization rates further underscores the clinical relevance of the platform-based workflow. Rehospitalization constitutes a robust outcome measure, reflecting complications severe enough to disrupt postoperative recovery and consume additional inpatient resources. The most pronounced reductions were observed in oncologic resection and reconstruction and in complex facial trauma, which are among the most resource-intensive domains of oral and maxillofacial surgery. Improved anatomical reproduction, accurate guide placement, and more predictable execution of planned osteotomies and reconstructions likely contributed to a lower incidence of postoperative events requiring readmission, consistent with observations from integrated digital workflow implementations in hospital-based POC environments [29].

Analysis of intraoperative plan modifications provides additional insight into workflow reproducibility and maturation. Intraoperative modifications represent a critical failure mode of preoperative planning and are often associated with increased operative time, greater tissue trauma, and elevated complication risk. The marked reduction in such modifications following adoption of the B-Onic platform indicates improved anticipation of surgical challenges during the preoperative phase. The clustering of modifications during the early adoption period highlights a learning curve effect, with stabilization occurring as imaging protocols, segmentation strategies, and validation workflows became standardized. This pattern mirrors previously reported experiences from hospital-based POC 3D printing laboratories operating under regulated quality systems [28,29].

Reductions in length of hospital stay and estimated intraoperative blood loss further illustrate how improved surgical precision translates into postoperative recovery and resource utilization. Length of stay decreased globally and showed the greatest reductions in oncologic and craniofacial or skull base procedures, paralleling reductions in complications and rehospitalization. Although length of stay is influenced by institutional discharge policies and non-clinical factors, the consistency of subgroup-specific improvements supports a genuine clinical effect. Estimated blood loss followed a coherent gradient, with significant reductions at the cohort level and in oncologic and craniofacial procedures, but not in trauma or orthognathic surgery. This distribution is surgically plausible, as precise osteotomy control and constrained dissection planes are most impactful in high-complexity procedures, whereas baseline blood loss in orthognathic surgery is typically low and less variable.

Beyond the interpretation of individual endpoints, this study contributes several novel elements to the scientific literature on digital surgery in oral and maxillofacial practice. First, it provides OMFS-only evidence within a platform-based ecosystem, avoiding the interpretative limitations inherent to multispecialty aggregation. Second, it evaluates a coordinated set of efficiency, reproducibility, and clinical outcome variables rather than isolated surrogate endpoints, offering a multidimensional assessment of clinical impact. Third, it bridges clinical outcomes with organizational and regulatory implementation principles derived from hospital-based POC manufacturing models, reinforcing the concept that digital technologies deliver maximal value when embedded within regulated, traceable workflows [8,28,29].

Several limitations should be acknowledged. The present study did not include a direct geometric accuracy or postoperative morphometric deviation analysis between planned and achieved surgical outcomes. Instead, surgical precision was evaluated indirectly through clinically relevant surrogate endpoints, including intraoperative plan modification rates, operative duration, complication incidence, rehospitalization, and blood loss. While these parameters reflect the practical reproducibility and execution fidelity of surgical plans, we acknowledge that they do not substitute for quantitative deviation analyses based on postoperative imaging or coordinate-based measurements. Future prospective studies incorporating standardized geometric accuracy metrics comparing planned and postoperative anatomical outcomes will be required to further characterize the precision performance of platform-based workflows. The retrospective design and use of historical controls introduce potential temporal confounding related to learning effects, incremental protocol refinements, and evolution of perioperative care. Although surgical teams and institutional pathways remained stable, these factors cannot be entirely excluded. Subgroup heterogeneity, particularly in trauma and oncologic reconstruction, may have diluted statistical power for certain endpoints despite clinically meaningful trends. Some outcome measures, such as estimated blood loss and length of hospital stay, are pragmatic and influenced by institutional practices and documentation standards. Finally, generalizability may be constrained by the availability of institutional engineering support and established quality management systems; adoption in smaller centers may require phased implementation and adaptation.

Future research should focus on prospective or stepped-wedge implementation designs, incorporation of standardized complexity metrics within oral and maxillofacial surgery subgroups, and formal health-economic analyses accounting for operating room time, bed-days saved, rehospitalizations avoided, and in-house versus outsourced manufacturing costs [1,2,6,8]. Long-term outcomes relevant to oral and maxillofacial surgery, including functional recovery, occlusal stability, facial symmetry, and patient-reported quality of life, should also be evaluated to fully define the value proposition of platform-based precision surgery [21,22,23,24,25,26,27,33,34,35,36]. In addition, continued development of immersive validation and planning tools based on extended reality may further enhance reproducibility and facilitate safe scaling across healthcare networks [30,31,32].

In summary, this OMFS-only analysis demonstrates that a regulated, platform-based point-of-care digital ecosystem is associated with clinically meaningful improvements in efficiency, reproducibility, and postoperative stability, particularly in high-complexity oral and maxillofacial procedures. By integrating clinical outcomes with organizational and regulatory implementation principles, this study advances the literature from isolated technological applications toward system-level precision surgery in routine oral and maxillofacial practice.

## 5. Conclusions

This study demonstrates that the implementation of a platform-based point-of-care digital ecosystem centered on the B-Onic platform can be effectively integrated into routine oral and maxillofacial surgical practice, providing consistent improvements across the perioperative pathway. By unifying medical imaging, virtual surgical planning, validation, and in-house additive manufacturing within a regulated workflow, the platform enabled significant reductions in preoperative planning time, validation time, and surgical duration.

Beyond workflow efficiency, the platform-based approach was associated with improved clinical outcomes, including lower postoperative complication rates, reduced unplanned rehospitalizations, shorter hospital stays, fewer intraoperative plan modifications, and decreased estimated intraoperative blood loss. These benefits were most evident in high-complexity procedures such as oncologic resection and reconstruction and craniofacial or skull base surgery, where surgical precision and reproducibility are critical. In contrast, highly standardized procedures such as orthognathic surgery showed more modest gains, reflecting optimized baseline outcomes.

By focusing exclusively on oral and maxillofacial surgery and adopting a pathology-based analytical framework, this study extends the existing literature beyond technology-centered reports and provides OMFS-specific evidence of the clinical and organizational value of integrated digital platforms. The findings support the incorporation of regulated, hospital-based point-of-care digital ecosystems as a viable strategy to enhance both surgical precision and healthcare efficiency in contemporary oral and maxillofacial practice.

Future prospective studies incorporating standardized complexity metrics, long-term functional outcomes, and formal health-economic evaluations are warranted to further define the role of platform-based point-of-care digital ecosystems. Nonetheless, the present results indicate that integrated and regulated digital platforms represent a practical and effective pathway toward precision surgery in oral and maxillofacial care.

## Figures and Tables

**Table 1:** Baseline demographic and clinical characteristics of the B-onic cohort and historical controls. Values are expressed as mean ± standard deviation or percentages.

Variable	B-Onic Group (*n* = 110)	Control Group (*n* = 72)
Number of cases	110	72
Mean age (years)	52.4 ± 15.1	54.1 ± 14.6
Sex (M/F, %)	67 (61%)/43 (39%)	42 (58%)/30 (42%)
Pathology-based subgroups (%)		
Facial trauma	34 (31%)	22 (30%)
Oncologic resection and reconstruction	32 (29%)	20 (28%)
Orthognathic surgery	29 (26%)	19 (27%)
Craniofacial or skull base procedures	15 (14%)	11 (15%)
ASA classification (III–IV) (%)	37 (34%)	26 (36%)
Elective procedures (%)	89 (81%)	57 (79%)

## Data Availability

The data presented in this study are available on request from the corresponding author.
